# Web-Based Health Information Following the Renewal of the Cervical Screening Program in Australia: Evaluation of Readability, Understandability, and Credibility

**DOI:** 10.2196/16701

**Published:** 2020-06-26

**Authors:** Olivia A Mac, Amy Thayre, Shumei Tan, Rachael H Dodd

**Affiliations:** 1 School of Public Health Faculty of Medicine and Health The University of Sydney Sydney Australia

**Keywords:** cervical screening, internet, consumer health information, Australia, papillomavirus infections

## Abstract

**Background:**

Three main changes were implemented in the Australian National Cervical Screening Program (NCSP) in December 2017: an increase in the recommended age to start screening, extended screening intervals, and change from the Papanicolaou (Pap) test to primary human papillomavirus screening (cervical screening test). The internet is a readily accessible source of information to explain the reasons for these changes to the public. It is important that web-based health information about changes to national screening programs is accessible and understandable for the general population.

**Objective:**

This study aimed to evaluate Australian web-based resources that provide information about the changes to the cervical screening program.

**Methods:**

The term *cervical screening* was searched in 3 search engines. The first 10 relevant results across the first 3 pages of each search engine were selected. Overall, 2 authors independently evaluated each website for readability (Flesch Reading Ease [FRE], Flesch-Kincaid Grade Level, and *Simple Measure of Gobbledygook* [SMOG] index), quality of information (Patient Education Materials Assessment Tool [PEMAT] for printable materials), credibility (*Journal of the American Medical Association* [JAMA] benchmark criteria and presence of Health on the Net Foundation code of conduct [HONcode] certification), website design, and usability with 5 simulation questions to assess the relevance of information. A descriptive analysis was conducted for the readability measures, PEMAT, and the JAMA benchmark criteria.

**Results:**

Of the 49 websites identified in the search, 15 were eligible for inclusion. The consumer-focused websites were classed as *fairly difficult to read* (mean FRE score 51.8, SD 13.3). The highest FRE score (easiest to read) was 70.4 (*Cancer Council Australia Cervical Screening Consumer Site*), and the lowest FRE score (most difficult to read) was 33.0 (*NCSP Clinical Guidelines*). A total of 9 consumer-focused websites and 4 health care provider–focused websites met the recommended threshold (sixth to eighth grade; SMOG index) for readability. The mean PEMAT understandability scores were 87.7% (SD 6.0%) for consumer-focused websites and 64.9% (SD 13.8%) for health care provider–focused websites. The mean actionability scores were 58.1% (SD 19.1%) for consumer-focused websites and 36.7% (SD 11.0%) for health care provider–focused websites. Moreover, 9 consumer-focused and 3 health care provider–focused websites scored above 70% for understandability, and 2 consumer-focused websites had an actionability score above 70%. A total of 3 websites met all 4 of the JAMA benchmark criteria, and 2 websites displayed the HONcode.

**Conclusions:**

It is important for women to have access to information that is at an appropriate reading level to better understand the implications of the changes to the cervical screening program. These findings can help health care providers direct their patients toward websites that provide information on cervical screening that is written at accessible reading levels and has high understandability.

## Introduction

### Background

In Australia, the latest figures for cervical screening from 2017 to 2018 show a 2-year participation rate of 53% for women aged 25 to 69 years [1] and an overall incidence of cervical cancer at 7 cases per 100,000 women [2]. Prevention of cervical cancer through the Australian National Cervical Screening Program (NCSP) was first introduced in 1991 and screened women aged 18 to 69 years every 2 years using cytology-based screening (Pap smear). The program was renewed in December 2017, and women aged 25 to 74 years are screened every 5 years using primary human papillomavirus (HPV) screening (Cervical Screening Test) [3]. This renewal was based on a greater understanding of the natural history of HPV and cervical cancer, successful uptake of the HPV vaccination leading to a subsequent reduction in vaccine-related HPV types, evidence that the HPV test is more sensitive than the Pap smear, and economic modeling demonstrating HPV screening to be more cost-effective [3]. Recent modeling studies have predicted that the new program will reduce the incidence and mortality of cervical cancer in vaccinated women by 31% and 36%, respectively [3,4]. A limited understanding of the rationale behind these changes has been demonstrated by the general population [5,6]. This highlights a need for information explaining these changes that is easy to understand and access to not undermine the confidence women have in the screening program. 

Almost 80% of Australians now use the internet as a source of health information, suggesting that the internet could be a powerful tool to educate and inform readers, with the potential to alleviate anxiety or concern [7]. As health models lean toward greater patient empowerment, patients may feel a greater sense of responsibility for their health care. However, because of the largely unrestricted nature of the internet and limited governance, there is a risk that users may be exposed to inaccurate, unreliable, biased, or potentially harmful information [8]. Exposure to information that is not presented in an accessible manner may cause unnecessary anxiety and distress. This could result in inappropriate care or people ignoring evidence-based recommendations because of being exposed to contradictory information [9].

Moreover, health information is only useful if it can be understood by the target population. The ability to read and understand written text is key for the comprehension of health information. Readability tools can measure the reading ability needed to understand the information presented. South Australia Health recommends that the readability level for health information is grade 8 (12-14 years old with 8 years of Australian education) [10,11]. In the United States, the National Institutes of Health and the American Medical Association recommend patient education materials to be written at or below the sixth-grade reading level (age 11 or 12 years), whereas the Joint Commission recommends a fifth-grade level (age 10 or 11 years) or lower [12]. There are limitations to using only readability measures to evaluate health care information [13], so it is important to ensure that web-based health information is of sufficient quality and is suitable to people from diverse backgrounds and varying levels of health literacy. Web-based health information may also require higher literacy levels than printed patient education materials, as previous studies have demonstrated that web-based patient education materials are often written above the recommended reading level of grades 6 to 8 [14-17], and these may be perceived as more difficult than print materials [13].

There is little to no value of health information that is trustworthy and credible if it cannot be easily understood and acted upon by the general population. A commonly cited disadvantage of web-based health information is the inability of consumers to evaluate the quality of websites [9,18]. In addition to being able to read health care information, consumers need to be able to understand the information presented, evaluate the credibility of websites, and find the information they are looking for.

### Objectives

To the best of our knowledge, no previous research has evaluated the readability of health information provided on websites about cervical screening in Australia, particularly regarding the changes to the NCSP. Therefore, this study aimed to evaluate the information available on the internet regarding the renewal of the NCSP in Australia, with a particular focus on website readability, understandability, design, credibility, and usability.

## Methods

### Identification of Websites

In April 2019, the term *cervical screening* was searched for in Australian versions of the 3 most popular search engines: *Google, Yahoo, and Bing*. The incognito window on Google Chrome was used to conduct the search, and browser history, cache, and cookies were cleared before running the search to ensure that previous searches would not impact the search results. The first 10 relevant results from the first 3 pages of results from each search engine were selected. Relevant websites (in the English language and related to cervical screening) were identified from each search engine, and any duplicate websites were removed and noted. Websites were excluded if they were advertisements, news reports, Wikipedia pages, social media pages, online discussion forums, blogs, videos, books, articles, or private websites.

### Measures

#### Readability

Readability was measured using Flesch Reading Ease (FRE), Flesch-Kincaid Grade Level (FKGL) [19], and the Simple Measure of Gobbledygook (SMOG) index [20].

##### Flesch Reading Ease and Flesch-Kincaid Grade Level

FRE uses a formula based on the average number of syllables, words per sentence, and the number of sentences to generate a readability score between 0 and 100 (Multimedia Appendix 1). Higher scores indicate greater ease of comprehension. A readability score above 60 is considered easy to follow by the general population [19]. The FRE has high reproducibility and correlation with other readability measures [21].

FKGL is a modified version of the FRE that generates the average US grade level required to understand the information. For example, an FKGL score of 8 indicates that the text can be understood by readers who have completed the equivalent of US grade 8 (Australian year 8; approximate age of 12-14 years) [19,22].

##### Simple Measure of Gobbledygook Index

The SMOG index assesses 10 consecutive sentences at the beginning, middle, and end of the relevant text and counts the number of polysyllabic words in each sentence [20]. These results are entered into a formula to establish the required grade level. A SMOG score of 3 to 8, 9 to 12, and 13 to 18 indicates that completion of primary, secondary, or tertiary education, respectively, is required to understand the information. For both FKGL and the SMOG index, a higher score indicates that a higher education level is required to understand the information.

The combination of our chosen readability measures (FRE, FKGL, and the SMOG index) is considered optimal, as they have been validated in the context of web-based health information and have high reliability for analyzing biomedical information [21].

#### Understandability and Actionability

The websites were evaluated for understandability and actionability using the validated Patient Education Materials Assessment Tool (PEMAT) for printable materials [23]. The PEMAT consists of 2 subscales: (1) understandability, which is a measure of the extent to which patient education materials can be understood by people of varying health literacy levels and diverse backgrounds; and (2) actionability, which measures how well a health consumer is able to identify what action to take based on the information provided [23]. Items are given a score of either 0 (disagree) or 1 (agree), with some items having a *not applicable* option. Final scores are calculated as a percentage of *agree* responses for all items, excluding those rated as *not applicable*. Higher percentages indicate higher understandability or actionability. A score higher than 70% indicates that materials are understandable and/or actionable [18]. As actionability was less relevant to this context, it was given less weight when determining the best overall website.

#### Website Credibility

##### Health on the Net Foundation Code of Conduct

The Health on the Net (HON) Foundation created a code of conduct that allows certified websites to display the Health on the Net Foundation code of conduct (HONcode) logo as a seal of approval [24]. The HONcode is recognized as an ethical code for websites and is based on 8 principles: authority, complementarity, privacy policy, attribution and date, justifiability, transparency, financial disclosure, and advertising policy. Website developers or information providers can apply for membership and request HONcode certification free of charge. The HONcode seal of approval is given to websites that comply with the principles mentioned earlier. Each website was evaluated for the presence of the HONcode logo.

##### Journal of the American Medical Association Benchmark

The *Journal of the American Medical Association* (JAMA) benchmark criteria enable the reader to easily discredit websites that lack reliability and transparency [25]. The criteria are as follows: (1) authorship—provides details about authors, contributors, affiliations, and credentials; (2) attribution—all references, sources, and copyright information to be provided; (3) currency—provision of the dates that content was updated; and (4) disclosure—website ownership, sponsorship, advertising policies, and potential conflicts of interest are prominently disclosed [25]. Websites were evaluated against each of the 4 benchmarks and given 1 point for each criterion met (final score 0-4).

#### Website Design

We used information on readability from the National Institute of Adult Continuing Education (NIACE) [26] to evaluate the design features that make the content of health care information more accessible and easier to understand (Multimedia Appendix 2). We analyzed the use of clear and distinct font styles (NIACE recommends fonts similar to Helvetica) and the adaptability of text (the ability to change the text size) and responsive web design (ie, the website displays differently according to the type of device, screen size, and orientation). Google Chrome features a *toggle device toolbar*, which allows the user to view how a website will be displayed on different screen sizes. We tested the responsiveness of each website by emulating different device types. We analyzed whether the font size appropriately reflected the purpose of the text (eg, larger text size for titles and headings). We also assessed whether the information was broken into *chunks* of text separated by white space as well as the presence and relevance of illustrations.

#### Usability: Simulation Questions

To emulate real life, 2 researchers, separately and independently, attempted to answer the following questions that women are likely to have related to the renewed NCSP: (1) Why did they change the interval from 2 to 5 years? (2) Why did they change the test? (3) Why did they change the age? (4) What are the benefits of the new test? and (5) Who should have the new test? Each researcher assessed whether the question was answered, and if yes, how well it was answered and how long it took to find the answer. The average time taken by both researchers was reported.

### Analysis

Readability statistics were obtained by copying and pasting each website URL into a web-based analysis tool, the *Readability Test Tool* [27], which uses an algorithm to calculate readability measures. If there were several pages on a website, statistics for each page were obtained, and the mean was calculated. An exception to this was if a website had a *publications and resources* page; the statistics for this page were not calculated. The analysis was stratified by the intended audience: consumer focused *versus* health care provider focused. Websites were categorized according to their primary target audience (ie, websites with most content aimed toward consumers were treated as consumer focused).

The quality of websites was evaluated by 2 independent researchers using Microsoft Excel. A third researcher independently coded 40% (6/15) of the websites. Descriptive statistics were calculated using IBM SPSS version 25. Means and SDs were calculated for understandability and actionability (PEMAT), readability statistics (FRE, FKGL, and SMOG), and JAMA benchmark criteria*.* Interrater reliability between the 2 reviewers was assessed using the Cohen kappa coefficient. Correlations between PEMAT and readability measures were calculated using the Pearson correlation coefficient. The Kruskal-Wallis test was conducted to detect differences in mean readability, understandability, actionability, and JAMA benchmark scores between consumer-focused and health care provider–focused websites.

### Ethical Approval

Ethical approval was not required, as the websites were in the public domain, and no human participants were involved.

## Results

### Search Results

**Figure 1:**
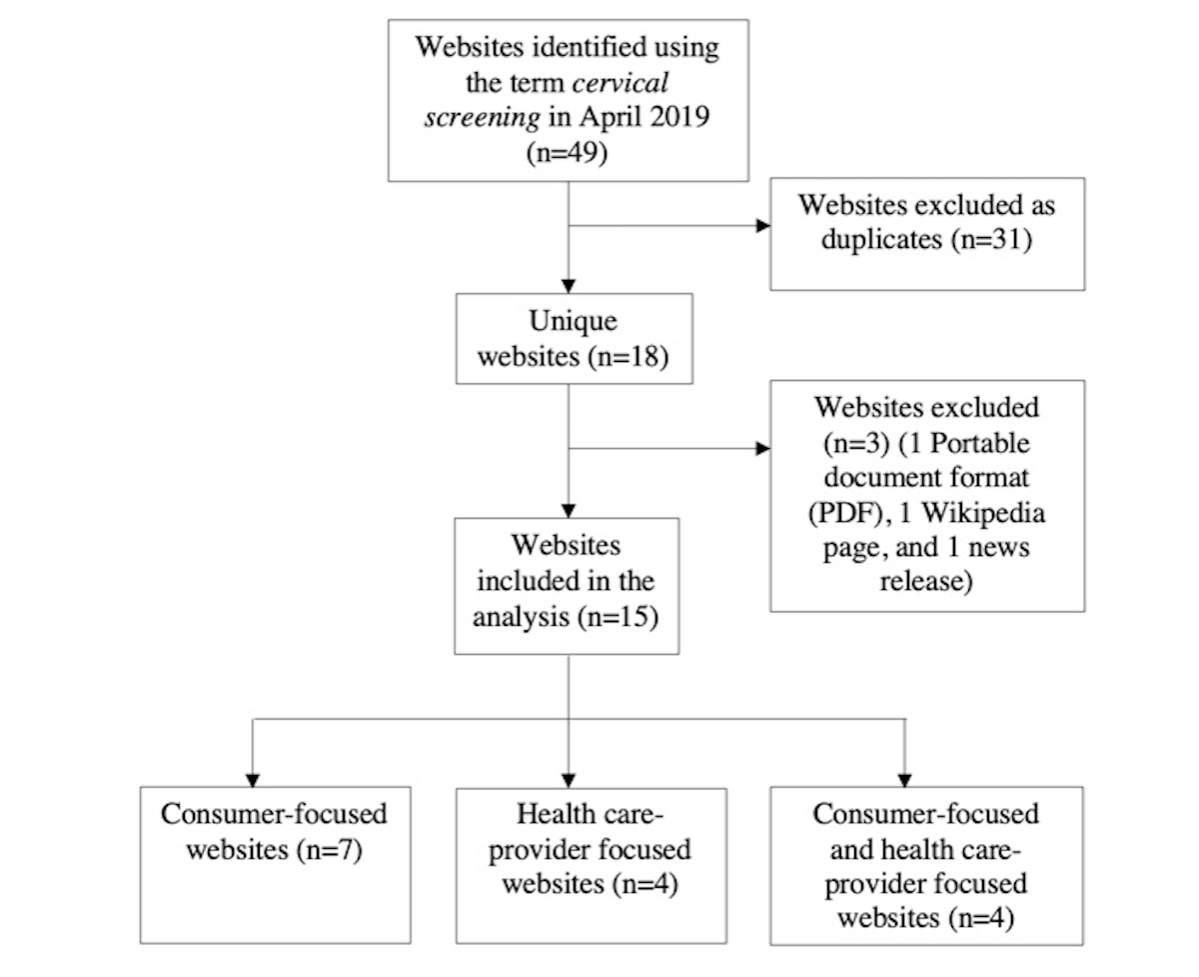
Flow diagram of inclusion and exclusion of websites for evaluation.

A total of 49 websites were identified using *Google* (n=16)*, Yahoo* (n=15), *and Bing* (n=18; Multimedia Appendix 3). After applying the exclusion criteria, 15 unique websites remained and were included in the evaluation (Figure 1). Duplicates (n=31) were excluded; therefore, a greater number of eligible websites were included from *Google*: *Google* (n=10), *Yahoo* (n=1), and *Bing* (n=4; full URLs can be found in Multimedia Appendix 3). The included websites were federal (*Cancer Australia)* or state government–owned (eg, *Cancer Institute NSW*), government-funded (*Health Direct)* or nongovernmental organizations (*Cancer Council).* Of the 15 included websites, 10 were targeted toward consumers, and 5 were targeted toward health care providers, including *National Prescribing Service (NPS) MedicineWise* that consisted of training modules for health care providers. In addition, 4 websites had information for both consumers and health care providers but were categorized according to the primary target audience. Abbreviations, target audience, and organizations of websites included in the analysis are described in Multimedia Appendix 4.

### Readability

The mean FRE of consumer-focused websites was 51.8 (SD 13.3; Table 1), which is considered *fairly difficult to read*. Health care provider–focused websites were considered *difficult to read*, with a mean FRE of 43.7 (SD 7.3). Of the consumer-focused websites, the *Cancer Council Australia Cervical Screening Consumer Site* had the highest FRE (70.4; Table 2), and the *Cancer Council Australia Main Site* had the lowest FRE (37.8). Consumer-focused websites had a mean reading grade level of 7.8 (SD 1.5), compared with a mean reading grade level of 8.1 (SD 1.4; Table 1) for health care provider–focused sites. *Health Direct* was the only website to score below the US-recommended sixth-grade level using the FKGL (Table 2). The mean SMOG index of consumer-focused sites was 6.7 (SD 1.2), compared with a mean SMOG index of 7.5 (SD 0.7; Table 1) for health care provider–focused sites. The SMOG index ranged from 4.6 (*Cancer Australia Cervical Screening)* to 9.1 *(Victoria Cervical Screening Program [CSP]).* Overall, 13 out of 15 (87%) of all included websites (9 consumer focused and health care provider–focused) scored between the sixth and eighth grade reading level using the SMOG index. *Victoria CSP* had the highest grade level (FKGL and SMOG) and the lowest reading ease (FRE). The differences in the FRE, FKGL, and SMOG index scores of consumer-focused and health care provider–focused websites were not statistically significant.

**Table 1:** Descriptive statistics of included websites by target audience.

Target audience of website		Flesch Reading Ease^a^ (0-100)	Flesch-Kincaid Grade Level^b^ (0-12)	SMOG^c^ index (3-18)	PEMAT^d^ understandability^e^ (0-100)	PEMAT actionability^e^ (0-100)	JAMA^f^ benchmark criteria^g^ (0-4)
**Consumers (n=10)**							
	Mean (SD)	51.8 (13.3)	7.8 (1.5)	6.7 (1.2)	87.7 (6.0)	58.1 (19.1)	2.4 (0.8)
	Median	50.0	7.6	6.8	90.0	60.0	2.0
	Range	36.4-70.4	5.9-10.4	4.6-9.1	75.0-93.0	20.0-93.3	1.0-4.0
**Health care providers (n=5)**							
	Mean (SD)	43.7 (7.3)	8.8 (1.1)	7.5 (0.7)	64.9 (13.8)	36.7 (11.0)	2.6 (1.3)
	Median	44.4	8.6	7.3	69.2	40.0	2.0
	Range	33.0-53.3	7.8-10.7	6.7-8.7	64.3-77.8	20.0-40.0	2.0-4.0
**All (n=15)**							
	Mean (SD)	48.9 (11.9)	8.1 (1.4)	7.0 (1.1)	79.0 (14.5)	51.0 (18.2)	2.3 (1.1)
	Median	45.0	8.2	6.9	83.6	85.0	2.0
	Range	33.0-70.4	5.9-10.7	4.6-9.1	64.3-93.0	20.0-60.0	1.0-4.0

^a^A Flesch reading ease score of 60 or higher is considered easy to read by the general public.

^b^Lower Flesch-Kincaid grade level and SMOG index indicate that content is easier to read. It is recommended that information is written between grades 6 and 8 (with grade 6 being easier to read).

^c^SMOG: Simple Measure of Gobbledygook.

^d^PEMAT: Patient Education Materials Assessment tool.

^e^A PEMAT score of 70 or higher indicates that content is understandable and/or actionable.

^f^JAMA: Journal of the American Medical Association.

^g^Scores represent the mean number of JAMA benchmark criteria satisfied. Websites were evaluated against each of the 4 benchmarks (authorship, attribution, currency, and disclosure) and given 1 point for each criterion met.

**Table 2:** Readability, Patient Education Materials Assessment Tool for Printable Materials (PEMAT-P) scores, and credibility of included websites.

Search engine and website			Readability							PEMAT^a^				Credibility		
			Flesch Reading Ease^b^(0-100)		Flesch-Kincaid Grade Level^c^ (0-12)		SMOG^d^ index^c^ (3-18)		Understandability^e^ (0-100)		Actionability^e^ (0-100)		HONcode^f^ presence (yes/no)		JAMA^g^ benchmark criteria (Au, At, D, and C)^h^	
**Google**																
	**Consumer focused**															
		NCSP^i^		60.6		6.9		6.9		93.0		83.3		No		D, C
		CCA^j^ main site		37.8		8.9		5.7		81.8		20.0		No		Au, At, D, C
		CCA cervical screening consumer site		70.4		6.3		6.8		85.7		60.0		No		Au, At, D
		Health Direct		68.1		5.9		6.6		92.3		40.0		Yes		At, D, C
		Jean Hailes		56.2		6.9		6.1		87.5		80.0		No		D, C
		WA^k^ CSP^l^		60.4		6.8		6.9		91.7		60.0		No		D, C
		NSW^m^ CSP		49.5		7.6		6.4		92.3		60.0		No		At
		Victoria CSP		36.4		10.4		9.1		90.0		60.0		No		At, C
		Queensland CSP (A)		44.4		8.2		6.7		69.2		40.0		No		D, C
	**Health care provider focused**															
		RACGP^n^		42.2		8.8		7.2		64.3		50.0		No		Au, At, D, C
**Yahoo**																
	**Consumer focused**															
		Cancer Australia cervical screening		40.7		8.1		4.6		75.0		60.0		No		Au, C
**Bing**																
	**Health care provider focused**															
		Queensland CSP (B)		39.2		8.8		6.5		82.4		60.0		No		D, C
		NPS^o^ MedicineWise		53.3		7.8		7.5		77.8		20.0		Yes		D, C
		NCSP clinical guidelines		33.0		10.7		8.7		71.4		33.3		No		Au, At, D, C
		NCSR^p^		45.5		8.6		7.3		41.7		40.0		No		Au

^a^PEMAT: Patient Education Materials Assessment Tool.

^b^A Flesch Reading Ease score of 60 or higher is considered easy to read by the general public.

^c^Lower Flesch-Kincaid Grade Level and SMOG index indicate content is easier to read. It is recommended that information is written between grade 6 and 8 (with grade 6 being easier to read).

^d^SMOG: Simple Measure of Gobbledygook.

^e^A PEMAT score of 70 or higher indicates content is understandable and/or actionable.

^f^HONCode: Health on the Net Foundation code of conduct.

^g^JAMA: Journal of the American Medical Association.

^h^Websites were evaluated against each of the 4 JAMA benchmark criteria: authorship (Au), attribution (At), disclosure (D), and currency (C).

^i^NCSP: National Cervical Screening Program.

^j^CCA: Cancer Council Australia.

^k^WA: Western Australia.

^l^CSP: cervical screening program.

^m^NSW: New South Wales.

^n^RACGP: Royal Australian College of General Practitioners.

^o^NPS: National Prescribing Service.

^p^NCSR: National Cancer Screening Register.

### Understandability and Actionability

The mean understandability score of consumer-focused websites was significantly higher than that of health care provider–focused websites (87.7%, SD 6.0% vs 64.9%, SD 13.8%; X^2^_1_=6.9; *P*=.01). Overall, 90% (9/10) of consumer-focused websites and 80% (4/5) of health care-provider focused websites met or exceeded the 70% threshold for understandability. The mean PEMAT actionability score was significantly higher in consumer-focused websites than in health care provider–focused websites (58.1%, SD 19.1% vs 36.7%, SD 11.0%; X^2^_1_=4.8; *P*=.04). Two consumer-focused websites met the threshold for actionability, whereas no health care provider-focused website was found to have actionable information. The *NCSP Main Site* and *Jean Hailes* were the only 2 websites to score over 70% in both the understandability and actionability domains. Interrater reliability was substantial for PEMAT ratings with 0.73 for understandability and 0.75 for actionability.

All 15 websites made their purpose evident and defined terms; however, only 13% (2/15; Table 3) of websites were judged as using visual aids whenever possible, and 53% (8/15) of the websites were judged to use the active voice*.* All consumer-focused websites, and 60% (3/5; Table 3) of health care provider-focused websites identified at least one action for the reader to take. Overall, 53% (8/15) of websites broke down the action into explicit steps, and 7% (1/15) of websites provided tangible tools whenever possible to help the reader take action. A correlation analysis was conducted between the FRE and PEMAT understandability to determine if websites that were easier to read were also easier to understand. There was no significant correlation found between reading ease and understandability.

**Table 3:** Percentage of *agree* responses on Patient Education Materials Assessment Tool (PEMAT) for Printable Materials items of the 15 included websites.

PEMAT^a^ items			Agree, n (%)^b^					
			Consumer-focused websites		Health care provider–focused websites		All websites	
**Understandability**								
	**Content**							
		Makes its purpose completely evident		10 (100)		5 (100)		15 (100)
		No distracting information		10 (100)		5 (100)		15 (100)
	**Word choice and style**							
		Common everyday language		10 (100)		3 (60)		13 (87)
		Medical terms are defined and used only to familiarize readers		10 (100)		3 (60)		13 (87)
		Active voice		6 (60)		2 (40)		8 (53)
	**Use of numbers**							
		Numbers are clear and easy to understand^c^		5(100)		3(75)		8 (73)
		Does not expect readers to do calculations		10 (100)		5 (100)		10 (100)
	**Organization**							
		*Chunks* information into short sections^d^		9 (100)		3 (100)		12 (100)
		Sections have informative headings^d^		9 (100)		3 (100)		12 (100)
		Presents information in a logical sequence		10 (100)		5(100)		15 (100)
		Provides a summary^d^		6 (67)		2 (67)		8 (67)
	**Layout and design**							
		Provides visual cues whenever possible		9 (90)		4 (80)		13 (87)
	**Use of VA^e^**							
		Uses VA whenever possible		2 (20)		0 (0)		2 (13)
		VA reinforce rather than distract^f^		4 (100)		1 (100)		5 (100)
		VA have clear titles and captions^f^		2 (50)		1 (100)		3 (60)
		VA are clear and uncluttered^f^		4 (100)		1 (100)		5 (100)
		Tables are simple with short, clear row and column headings^g^		3 (100)		1 (100)		4 (100)
**Actionability**								
	Identifies at least one action for the user		10 (100)		3 (60)		13 (87)	
	Addresses the user directly		9 (90)		3 (60)		11 (73)	
	Breaks down actions into explicit steps		6 (60)		2 (40)		8 (53)	
	Provides tangible tools whenever it could help		0 (0)		1 (20)		1 (7)	
	Instructions and examples for calculations^h^		N/A^i^		N/A		N/A	
	Explains how to use the charts, diagrams etc^j^		2 (100)		0 (0)		2 (67)	
	Uses VA whenever possible to help take action		2 (20)		2 (40)		4 (27)	

^a^PEMAT: patient education materials assessment tool.

^b^Agree (%) was calculated by the following formula: total number of *agrees*/total number of applicable websites.

^c^Not applicable for 4 websites.

^d^Not applicable for 3 websites.

^e^VA: visual aids.

^f^Not applicable for 10 websites.

^g^Not applicable for 11 websites.

^h^Not applicable for all 15 websites.

^i^N/A: not applicable.

^j^Not applicable for 12 websites.

### Credibility: Journal of the American Medical Association Benchmark Criteria and Health on the Net Foundation Code of Conduct

Overall, 3 websites met all 4 of the JAMA benchmark criteria (*Cancer Council Australia Main Site*, *NCSP Clinical Guidelines*, and *the Royal Australian College of General Practitioners [RACGP]).* The mean number of JAMA benchmark criteria satisfied was 2.4, ranging from 1 to 4. Overall, currency was the most well-adhered principle, with 8 of 10 consumer-focused and 4 of 5 health care provider–focused websites providing update information (Table 4). Authorship was the most commonly missed criteria, with only 3 consumer-focused and 3 health care provider–focused websites properly attributing the authors of information, including their qualifications and affiliations. There was no significant difference in the mean number of criteria satisfied between consumer-focused and health care provider–focused websites.

The HONcode was only present on 2 websites (*NPS MedicineWise* and *Health Direct*; Table 2). No websites displayed an explicit conflict of interest statement (represented as part of the disclosure criteria); therefore, the existence of any actual or potential conflicts cannot be determined. *RACGP* provided a link to a conflict of interest management policy, which states that no one with a conflict of interest can be involved in the decision-making process. *NCSP Clinical Guidelines* provided a register of all interests, leaving it up to the end user to determine the presence and significance of any conflict.

**Table 4:** Number of included websites adhering to the Journal of the American Medical Association benchmark criteria.

JAMA^a^ benchmark criteria	Websites adhering, n (%)		
	Consumer-focused websites	Health care provider–focused websites	All websites
Authorship	3 (30)	3 (60)	6 (40)
Attribution	6 (60)	2 (40)	8 (53)
Disclosure	7 (70)	4 (80)	11 (73)
Currency	8 (80)	4 (80)	12 (80)

^a^JAMA: Journal of the American Medical Association.

### Website Design

All websites had consistent use of white space, separating paragraphs of information into smaller *chunks* of text. All websites used distinct and easy-to-read font styles and had clear subheadings in a text size larger than the main text. An adaptable font size was available in 2 of 15 websites (*Cancer Council Australia Main Site* and *Cancer Australia Cervical Cancer)*. All 15 websites used a responsive web design. Of 15 websites, 3 (*NSW CSP, Cancer Council Australia Cervical Screening Consumer Site,* and *Jean Hailes)* used illustrations to reinforce key messages.

### Simulation Questions

Of 15 websites, 3 (*NCSP Main Site, Cancer Council Australia Cervical Screening Consumer Site,* and *NSW CSP*) provided answers to all 5 simulation questions*.* The question most frequently answered by websites was question 5: “Who needs the new test?” *NPS MedicineWise, RACGP, National Cancer Screening Register,* and *Queensland CSP (B)* did not provide answers to any of the questions. The time taken to find the answers ranged from approximately 10 seconds to 2 min. The answers were found the fastest on *NSW CSP* (average time of 34 seconds per question) and the slowest on *NCSP Clinical Guidelines* (average time of 146 seconds per question).

## Discussion

### Principal Findings

This study is the first to evaluate web-based information available to consumers about the renewal of the Australian NCSP. Overall, the readability of consumer-focused websites providing information about the renewed NCSP was fairly difficult to read by the general population (ie, below the threshold of 60); however, there was high variability in scores. Overall, the evaluated websites demonstrated a high level of understandability. The *Cancer Council Australia Cervical Screening Consumer Site* scored highest for understandability assessed using the PEMAT. There was no significant correlation between reading ease and understandability, demonstrating that they measure different constructs. This is in line with previous studies and highlights the importance of using both measures to evaluate quality [17]. Most websites met some criteria to assess their credibility, and all websites demonstrated some thought had been given to the design of the websites. Of the 5 key questions that could be frequently searched by consumers, the following 3 websites provided answers to all questions: *NCSP Main Site, Cancer Council Australia Cervical Screening Consumer Site,* and *NSW CSP.* These findings demonstrate great variability in the readability, understandability, and credibility of websites available on the internet, which provide both consumers and health care providers with information about the changes to the NCSP.

Across all measures, the *Cancer Council Australia Cervical Screening Consumer Site* was judged to be the most accessible website overall. This site was considered the easiest to read based on the FRE score and had a reading level between grade 6 and 7 using both the FKGL and SMOG index, which is in line with recommendations from South Australia Health [11]. Furthermore, it scored highly on the understandability criterion of the PEMAT and was one of only three websites to answer all 5 simulation questions. This website was developed by Cancer Council Australia in response to the renewal of the NCSP and has a clear purpose to educate women about the changes to cervical screening and seeks to answer possible frequently answered questions. It provides comprehensive content about the changes in an accessible manner. Australian women with questions or concerns about the renewed NCSP may benefit from being directed to this website by their health care provider.

### Strengths and Limitations

This study used validated tools and a combination of several measures to independently evaluate websites providing information about cervical screening. Objective readability measures provide limited data about the health literacy level required to understand information, so by combining this with the PEMAT, this enabled us to determine how well people of various backgrounds and levels of health literacy may be able to understand and act upon the provided health information. These tools were selected because the items were most relevant to the health information we were evaluating. Other available tools, such as DISCERN [28], have a number of items aimed at treatment-related information, which are not relevant for the content of cervical screening information*.* A limitation of our study is the small number of websites included in the analysis, which may have prevented seeing differences in readability between health care provider–focused and consumer-focused websites. However, the search term *cervical screening* returned over 50,000,000 results on Google alone, and as internet users rarely look past the first 3 results pages, it is likely that the websites included in our analysis would be those most commonly accessed by consumers [18]. Some of the measures we used are subjective (eg, PEMAT), but by having 2 independent evaluators score all 15 websites and a third evaluator score 40% (6/15) of websites, this will have helped ease any discrepancies, and the interrater reliability between the evaluators was rated as substantial. The websites in this study were only examined using their content and were not examined for accuracy of the information presented, as this was not an aim of the study. This study was limited to the evaluation of printable material on the websites; therefore, information delivered in video format was excluded.

### Comparison With Previous Work

This study is particularly relevant in light of the renewed NCSP and the public misconception about the rationale behind the changes [5,6]. Our findings show that most consumer-focused web-based information on cervical screening in Australia is written at a reading level inaccessible to the general population. This is similar to previous studies that have found web-based health information to often be written above the recommended reading level [29]. This may be because the authors have insufficient awareness of health literacy considerations and readability [30].

In this study, websites with the lowest readability tended to be those targeted toward health care providers. Making the target audience of the website more explicit may help avoid potential confusion for consumers and the frustration of not having questions answered. Although this may be obvious for some websites (eg, *NCSP Clinical Guidelines*), health consumers should be able to determine at a glance the target audience of the information they are reading for all websites*.* This may minimize their exposure to inaccessible material. Sites aimed toward health care providers could provide links to guide consumers to reputable consumer-focused websites (eg, *NCSP Main Site* or *Cancer Council Australia Cervical Screening Consumer Site*)*.*

The high understandability scores indicate that web-based cervical screening information can be understood relatively easily by consumers of diverse backgrounds and levels of health literacy [23] and demonstrate that the PEMAT and readability tools measure different constructs. Greater awareness of how the mode of delivery may affect consumers’ understanding of the health information could help to create and distribute better patient materials. The low actionability scores suggest that it may be difficult for consumers to act upon the information they read. Most websites failed to provide tangible tools or visual aids and lacked explicit instructions for the user to follow (eg, a tool to help consumers decide whether they are eligible for screening under the new guidelines). The general purpose of these websites is to inform and educate the reader about the changes rather than encouraging an action. Consequently, the actionability domain of the PEMAT may have limited applicability to this context. The large range in PEMAT scores may be partially explained by the fact that some websites were solely targeted toward health care providers. As such, the content on these websites (*NCSP Clinical Guidelines, RACGP,* and *NPS MedicineWise*) would not be considered *patient* education materials, and consequently, the PEMAT score may not be an accurate measure of quality. Most existing literature on web-based health information is related to disease and treatment information, where there may be more explicit actions to take than for cervical screening (eg, different treatment options for a chronic disease). This may, in part, contribute to the low actionability scores for our websites, but because of actionability not being an aim of these websites, less weight was given to these scores.

Noting the presence of the HONcode logo is a simple way for consumers to recognize that the information they are reading is trustworthy, but because HONcode certification is a voluntary registration process, it is possible that websites may comply with the principles despite not being certified [10]. As most of the included websites are federal or state government owned, they would likely be instantly recognized as trustworthy and credible sources of information; therefore, there may be no added benefit of HONcode certification. It is unclear how recognizable the HONcode is among both consumers and website developers. The only 2 websites displaying the HONcode are government-funded not-for-profit organizations (*Health Direct* and *NPS Medicine Wise)*. As these sites may not be as easily recognized as reputable sources, HONcode certification may be more valuable; however, there is little value if a consumer does not know what it is or how to interpret it.

Most websites failed to provide adequate detail about the authors of the information or to provide sources and references for information. This information was seldom displayed prominently, often located on the *about us* or *disclaimer* pages, which are rarely accessed by consumers [18]. Sources that were provided were rarely primary research and often linked to related websites, raising the potential of consumers to be exposed to inaccurate, inaccessible, or unreliable content if the linked websites are of poor quality or lack credibility. It could be argued, however, that the omission of primary sources on consumer-focused websites may be a deliberate decision by the website developer. Primary research papers are often behind paywalls or written at a reading level inaccessible to the general public. This has the potential to cause greater confusion and may offer little benefit to the reader. Furthermore, consumers generally judge the credibility of websites based on the hosting organization (ie, whether it is from a reputable and recognizable organization such as the Australian Government or Cancer Council Australia), language, and professional-looking design [18]. Web-based information can be biased, with little to no scientific evidence [18]. In addition to providing credit to the original source, appropriate attribution can help the end user evaluate the quality and ensure the trustworthiness of information on the web. This leads to a discussion of whether there should be mandatory criteria to follow when setting up websites to ensure their credibility. Furthermore, it raises the question of whether details about sources, authors, and conflicts of interest can be displayed in a way that is more accessible and meaningful to health consumers.

The 3 websites that satisfied all 4 JAMA benchmarks were targeted specifically toward health care providers. If websites targeted toward health consumers lack the aforementioned principles, it raises potential issues. The general population, particularly those of lower health literacy, may be less equipped than health care providers to evaluate and discredit websites. It is critical that consumer-focused websites are credible, but it may be necessary for a tradeoff between accessibility, readability, understandability, and credibility. This area requires further research to minimize this tradeoff, as each component contributes toward consumers’ experience of accessing health information on the web. Appropriate attribution and references are a particularly important credibility indicator of health care provider–focused websites; however, they may be considered less relevant for consumer-focused sites. Existing credibility assessment tools (such as the JAMA benchmark criteria and the HONcode criteria) are limited in their ability to evaluate consumer-focused websites. The development of a new credibility framework for consumer-focused websites could mitigate this limitation. Taking into consideration other factors that influence credibility, such as placing more weight on the hosting organization, could enable consumers to discredit unreliable information without reducing the overall quality.

Owing to an increasing interest in the literature about conflicts of interest, it was interesting to find that none of the websites in this study explicitly disclosed a conflict of interest. It is important to note that the absence of a conflict of interest statement is not equivalent to having no conflict. There is increasing research into the management of conflicts of interest and whether or not disclosure is the most appropriate strategy [31]. Disclosing conflicts merely indicates the possibility of a bias; however, it does not provide guidance for resolving it or how the conflict was managed. For the consumer, a lack of disclosure may hinder their ability to mitigate any potential risk of bias, as conflicts of interest may impact decision making [31].

Simulation questions were helpful in establishing whether these websites explained the changes to the CSP. The majority of these websites did not answer the questions in detail, raising concerns about whether consumers reading the information would be fully informed about the changes. A total of 3 websites provided answers to all the questions (*NCSP Main Site, Cancer Council Australia Cervical Screening Consumer Site,* and *NSW CSP*), with the time taken to find answers to these questions being the quickest for the *NCSP Main Site.* The websites that either did not provide answers to the simulation questions or it took a long time to find the answers were those aimed toward health care providers, further highlighting the need for the target audience of the website to be made more explicit.

Finally, all websites were designed to improve the accessibility of information through the use of white space and appropriate font style. The layout and design of materials can be a contributing factor to how consumers comprehend the information or how accessible the information is [22]. Only 2 websites showed adaptable font size, which may cause potential difficulties for those with impaired sight. An important consideration when designing websites now is also about how the interface looks on mobile phones, given the increasing number of consumers who will view health information on their mobile phones [7]. It was therefore reassuring to observe that all websites used responsive web design. The use of illustrations can reinforce messages and aid interpretation of written text or they can distract away from the purpose [26]. The *NSW CSP* and *Cancer Council Australia Cervical Screening Consumer Site* use photographs of culturally and demographically diverse women, which may help consumers understand that the information is relevant to them.

### Conclusions

The findings from this study can help health care providers direct their patients toward websites that have information about the renewal of the CSP, which is easy to read and has high understandability (eg, *Cancer Council Australia Cervical Screening Consumer Site*). Encouragingly, web-based information about the renewed CSP is generally of good quality. There is a need to promote increased awareness of the importance of web-based health information that is credible, user friendly, and easily understood by people with wide levels of health literacy. Web-based information should be produced with particular consideration to people with low health literacy, with those responsible for creating these websites to be accountable for ensuring that this information is accessible, accurate, and credible. There may be potential for web-based health information to be improved through the introduction of mandatory criteria to ensure the credibility and quality of consumer-focused websites.

## Appendix

Multimedia Appendix 1 Description of website evaluation measures.

Multimedia Appendix 2 National Institute of Adult Continuing Education tool, Readability: producing clear written materials for a range of readers.

Multimedia Appendix 3 Website URLs of all identified websites by search order.

Multimedia Appendix 4 Website abbreviations, organisation and target audience of included websites.

## Abbreviations

CSP: Cervical Screening Program
FKGL: Flesch-Kincaid Grade level
FRE: Flesch Reading Ease
HONcode: Health on the Net Foundation code of conduct
HPV: human papillomavirus
JAMA: Journal of the American Medical Association
NCSP: National Cervical Screening Program
NIACE: National Institute of Adult Continuing Education
PEMAT: Patient Education Materials Assessment tool
RACGP: Royal Australian College of General Practice
SMOG: Simple Measure of Gobbledygook index

## References

[1] (2019). Australian Institute of Health and Welfare.

[2] (2019). Australian Institute of Health and Welfare.

[3] (2013). Cancer Screening.

[4] Lew JB, Simms KT, Smith MA, Hall M, Kang YJ, Xu XM, Caruana M, Velentzis LS, Bessell T, Saville M, Hammond I, Canfell K (2017). Primary HPV testing versus cytology-based cervical screening in women in Australia vaccinated for HPV and unvaccinated: effectiveness and economic assessment for the national cervical screening program. Lancet Public Health.

[5] Obermair HM, Dodd RH, Bonner C, Jansen J, McCaffery K (2018). 'It has saved thousands of lives, so why change it?' Content analysis of objections to cervical screening programme changes in Australia. BMJ Open.

[6] Dodd RH, Obermair HM, McCaffery KJ (2019). A thematic analysis of attitudes toward changes to cervical screening in Australia. JMIR Cancer.

[7] Cheng C, Dunn M (2017). How well are health information websites displayed on mobile phones? Implications for the readability of health information. Health Promot J Austr.

[8] Cocco AM, Zordan R, Taylor DM, Weiland TJ, Dilley SJ, Kant J, Dombagolla M, Hendarto A, Lai F, Hutton J (2018). Dr Google in the ED: searching for online health information by adult emergency department patients. Med J Aust.

[9] Sun Y, Zhang Y, Gwizdka J, Trace CB (2019). Consumer evaluation of the quality of online health information: systematic literature review of relevant criteria and indicators. J Med Internet Res.

[10] (2011). The Australian Curriculum, Assessment and Reporting Authority.

[11] (2013). Australian Commission on Safety and Quality in Health Care.

[12] Grabeel KL, Russomanno J, Oelschlegel S, Tester E, Heidel RE (2018). Computerized versus hand-scored health literacy tools: a comparison of simple measure of gobbledygook (SMOG) and Flesch-Kincaid in printed patient education materials. J Med Libr Assoc.

[13] Farnsworth M (2014). Differences in perceived difficulty in print and online patient education materials. Perm J.

[14] Nghiem A, Mahmoud Y, Som R (2016). Evaluating the quality of internet information for breast cancer. Breast.

[15] Lipari M, Berlie H, Saleh Y, Hang P, Moser L (2019). Understandability, actionability, and readability of online patient education materials about diabetes mellitus. Am J Health Syst Pharm.

[16] Kirby PL, Reynolds KA, Walker JR, Furer P, Pryor TA (2018). Evaluating the quality of perinatal anxiety information available online. Arch Womens Ment Health.

[17] Aguirre PE, Coelho MM, Rios D, Machado MA, Cruvinel AF, Cruvinel T (2017). Evaluating the dental caries-related information on Brazilian websites: qualitative study. J Med Internet Res.

[18] Eysenbach G, Köhler C (2002). How do consumers search for and appraise health information on the world wide web? Qualitative study using focus groups, usability tests, and in-depth interviews. Br Med J.

[19] Flesch R (1948). A new readability yardstick. J Appl Psychol.

[20] McLaughlin GH (1969). SMOG grading: a new readability formula. J Read.

[21] Beaunoyer E, Arsenault M, Lomanowska AM, Guitton MJ (2017). Understanding online health information: evaluation, tools, and strategies. Patient Educ Couns.

[22] Shedlosky-Shoemaker R, Sturm AC, Saleem M, Kelly KM (2009). Tools for assessing readability and quality of health-related web sites. J Genet Couns.

[23] Shoemaker SJ, Wolf MS, Brach C (2014). Development of the patient education materials assessment tool (PEMAT): a new measure of understandability and actionability for print and audiovisual patient information. Patient Educ Couns.

[24] Boyer C, Selby M, Scherrer J, Appel R (1998). The health on the net code of conduct for medical and health websites. Comput Biol Med.

[25] Aday LA (1997). Benchmarks of fairness for health care reform. J Am Med Assoc.

[26] (2009). Learning and Work Institute.

[27] WebFX.

[28] Charnock D, Shepperd S, Needham G, Gann R (1999). DISCERN: an instrument for judging the quality of written consumer health information on treatment choices. J Epidemiol Community Health.

[29] Cheng C, Dunn M (2015). Health literacy and the Internet: a study on the readability of Australian online health information. Aust N Z J Public Health.

[30] Boztas N, Omur D, Ozbılgın S, Altuntas G, Piskin E, Ozkardesler S, Hanci V (2017). Readability of internet-sourced patient education material related to 'labour analgesia'. Medicine (Baltimore).

[31] Dunn AG, Coiera E, Mandl KD, Bourgeois FT (2016). Conflict of interest disclosure in biomedical research: a review of current practices, biases, and the role of public registries in improving transparency. Res Integr Peer Rev.

