# Supplementary material for: Web-Based Health Information Following the Renewal of the Cervical Screening Program in Australia: Evaluation of Readability, Understandability, and Credibility
Source: J Med Internet Res. 2020 Jun 26;22(6):e16701. doi: 10.2196/16701 (PMC7381085; doi:10.2196/16701)
Supplement: Multimedia Appendix 1 [file jmir_v22i6e16701_app1.pdf]

Multimedia appendix: Description of website evaluation measures.

	Description	Formula	Interpretation
**Readability**			
**Flesch Reading Ease (FRE)**	FRE calculates the approximate reading level of English-language content based on average sentence length and words per sentence. A reading ease above 60 is considered easy to read by the general population.	RE = 206.835 – (1.015 x ASL) – (84.6 x ASW)	90-100: Very easy<br>80-89: Easy<br>70-79: Fairly Easy<br>60-69: Standard<br>50-59: Fairly Difficult<br>30-49: Difficult<br>0-29: Very Confusing
**Flesch Kincaid Grade Level (FKGL)**	FKGL calculates the approximate U.S grade level required to read a text. It is recommended that text aimed towards the general public should have a grade level of around 8.	(0.39 x ASL) + (11.8 x ASW) = 15.59	A score of 8.0 indicates that the average reader needs a reading level equivalent to U.S grade 8 (approximate age 12-14). The lower the FKGL, the easier the content is to read.
**SMOG Index**	SMOG index estimates the number of years in education a person needs in order to understand a piece of text based on the density of complex words. SMOG index is recommended for assessing readability of health information as it predicts 100% comprehension.	3 + square root of the polysyllable count	A SMOG index of 8 indicates 8 years of education are required (e.g. a U.S grade level of 8). The lower the SMOG index the easier the content is to read.<br><br>3-8: primary education required<br>9-12: secondary education required<br>13-18: tertiary education required.
**Patient Education Material Assessment Tool (PEMAT)**			
**Understandability**	The understandability domain measures how well consumers of diverse backgrounds and levels of health literacy can understand patient education material.	19 items for understandability are rated ‘agree’ or ‘disagree’ with ‘not applicable’ available for some items. Scores are calculated as a percentage of ‘agree’ responses out of all possible points (i.e.	Patient education materials with a score of 70% or over are considered easy to understand by people diverse backgrounds and varying levels of health literacy. The higher the score, the easier the information is to understand.

		excluding those scored NA).	
**Actionability**	The actionability domain assesses how well consumers can identify what to do based off the information they have read.	7 items for actionability are rated ‘agree’ or ‘disagree’ with ‘not applicable’ available for some items. Scores are calculated as a percentage of ‘agree’ responses out of all possible points (i.e. excluding those scored NA).	Patient education materials are considered actionable if they score 70% or over. High scores indicate more actionable information.
**Credibility**			
**JAMA benchmark criteria**	Four criteria that assess the presence of authorship, attribution currency and disclosure of health information.	The presence of each criteria is assessed, and the number of benchmarks met is reported.<br>*Authorship*: detail about authors, credentials and qualifications<br>*Attribution*: references, sources and copyright information<br>*Disclosure*: advertising policy, conflict of interest policy, website ownership,<br>*Currency*: data of posted and updated information	The JAMA benchmark criteria ranges from 0-4. A higher score indicates the website is more credible.
**HONCode**	The HONCode is a voluntary certification seal for medical and health related information. Accreditation is given to applying websites that meet the principles of authority, complementarity, confidentiality, attribution, justifiability, transparency and financial disclosure.	Websites are checked for the presence of the HONcode seal. Presence of the HONcode indicates that the websites adhere with all 8 principles.<br>*Authority:* provide qualifications of authors<br>*Complementarity:* information is intended to support not replace information from a healthcare professional.<br>*Confidentiality:* privacy of user’s data is respected.	The presence of the HONcode seal indicates the information is trustworthy and adheres to the HONcode of conduct.

		Attribution: information supported by references.<br>*Justifiability:* any claims supported by appropriate evidence.<br>*Transparency:* provides valid contact details.<br>*Financial disclosure:* details of funding are provided<br>*Advertising:* details about advertising and policy is clear and distinct.	

FRE: Flesch Reading Ease, RE: readability ease, ASL: average sentence length (number of words divided by number of sentences), ASW: average number of syllables per word, FKGL: Flesch Kincaid Grade Level, SMOG: Simple Measure of Gobbledygook, PEMAT: Patient Education Material Assessment Tool, JAMA: Journal of American Medical Association HONcode: Health on The Net Code

This is a Multimedia Appendix to a full manuscript published in the J Med Internet Res. For full copyright and citation information see http://dx.doi.org/10.2196/jmir.16701
