# Supplementary material for: Web-Based Health Information Following the Renewal of the Cervical Screening Program in Australia: Evaluation of Readability, Understandability, and Credibility
Source: J Med Internet Res. 2020 Jun 26;22(6):e16701. doi: 10.2196/16701 (PMC7381085; doi:10.2196/16701)
Supplement: Multimedia Appendix 2 [file jmir_v22i6e16701_app2.pdf]

Readability

How to produce clear written materials for a range of readers

niace
promoting adult learning

For adults and young people to participate fully in work, learning and everyday activities, it is essential that they are able to read and understand written information.

This increasingly demands a high level of comprehension and the application of knowledge, rather than a simple ability to recognise letters and decode words.

People who lack confidence in these crucial skills are often faced with an immediate barrier if the written information they are given doesn't take account of their difficulties.

It's possible to present written information in a way that makes reading easier, as part of a strategy to address this issue.

Both the design and the readability levels of texts determine how easy they are to read.

This leaflet outlines the principles that will help you to present written texts clearly, taking account of the needs of your readers.

1. Design

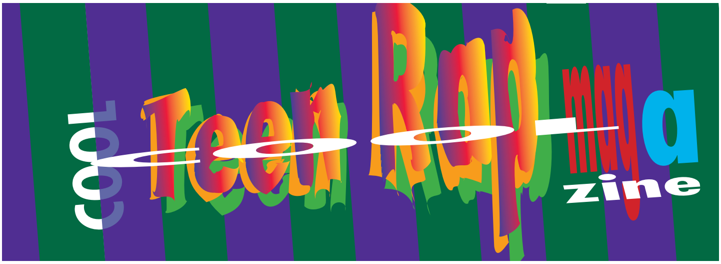

Difficulties with reading are often more to do with the look and layout of a text than with the complexity of the text itself. In their efforts to produce attractive, eye-catching material, designers are sometimes tempted to sacrifice clear layout. Complicated layout and design can confuse those with reading difficulties. Here are some of the elements to watch for.

• White space

It's important that readers can find their way around a text easily. Too much text on a page can be a deterrent to getting started on reading. Columns too close together can cause confusion, leading to reading 'over' from one to the next.

Anyone with reading problems needs short, clearly separated 'chunks' of text that they can work through at their own pace. This helps them to see how far they have to go, and reduces the chances of them giving up. Pages that have no margins, or little space between paragraphs, are generally more difficult to read.

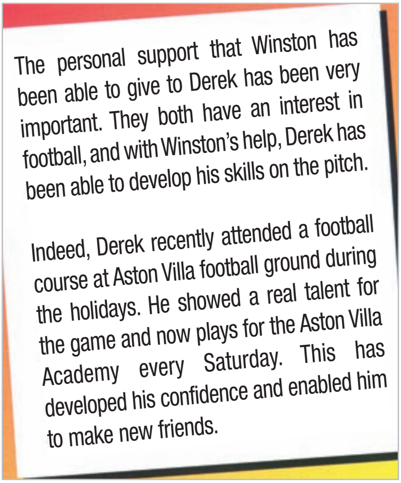

The personal support that Winston has been able to give to Derek has been very important. They both have an interest in football, and with Winston's help, Derek has been able to develop his skills on the pitch.

Indeed, Derek recently attended a football course at Aston Villa football ground during the holidays. He showed a real talent for the game and now plays for the Aston Villa Academy every Saturday. This has developed his confidence and enabled him to make new friends.

• Line spacing

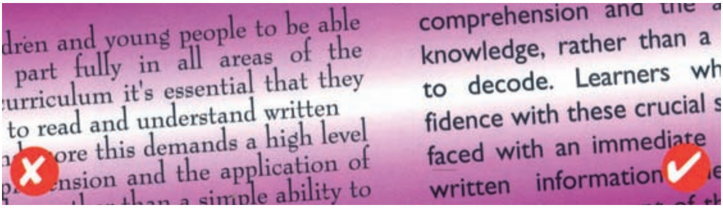

The spacing between lines is also an important factor in making reading easier. If they are too close the hesitant reader will tend to drop lines; when they are too far apart the reader will not be clear whether the lines relate to each other at all. Line spacing (leading) depends on type size but with normal 12pt type, a leading of 2pt is sensible.

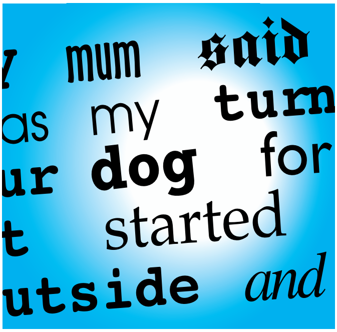

• Font choice and size

There are endless debates about whether serifed or sans serifed types are easier to read and whether those with reading difficulties find it hard to recognise 'a' or 'g' in the different types. Most people with reading difficulties can recognise and differentiate the letters of the alphabet. Types chosen need to be reasonably clear (Gothic not recommended, for example), and distinct (avoid types where 'rn' can easily be mistaken for 'm', etc.). Much material is set in Century Schoolbook, Plantin or Helvetica, although *Comic Sans* is also popular. Arial is a good choice for documents created on a word processor.

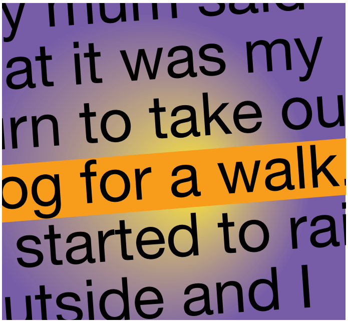

Type size inevitably relates to the nature and purpose of the text.

It is worth watching for too large a type for booklets or leaflets produced for older readers. They may be put off if they feel the material appears to be childish.

• Use of upper and lower case

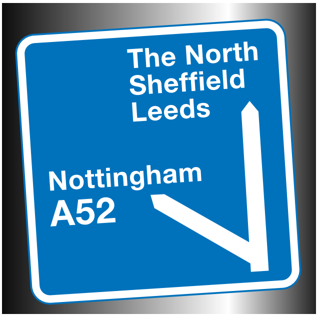

You may have noticed that major road signs use upper and lower case for cities and towns, whereas on minor roads the older signposts still show directions in upper case only. Upper and lower case is easier to read (for all of us) than upper case only. The shape of the word is an aid to the reading of the name of the town (e.g. The North, Sheffield, Leeds and Nottingham, as opposed to SHEFFIELD or LEEDS).

The same is true for reading texts: the overuse of upper case, for example to convey emphasis, is counter-productive. It is less likely that the text will be read, not more likely. Far better to use **bold type**, or boxing , to show the importance of a part of the text.

• Illustrations and overprinting

Illustrations and photographs break up the density of text. They work best if they relate directly to the surrounding text, so that a learner with reading difficulties can use the illustration as a clue to the text itself. The illustration should, wherever possible, come at the end of paragraphs or sentences, rather than in the middle of them.

Illustrations are sometimes used as background, with print running over them. This generally makes the text more difficult to read.

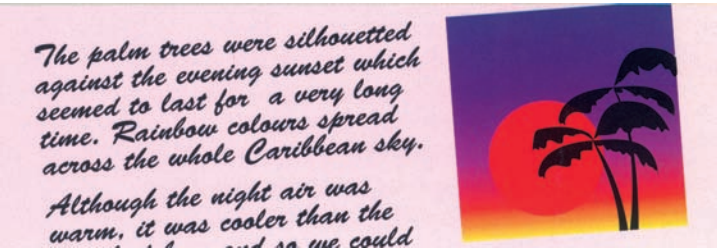

• Page layout and page breaks

In addition to the use of white space, look at the layout of pages. Ideally headings and new sections should come at the top of pages, and sentences and paragraphs should not run over columns or pages. Lines between columns can be helpful. Page numbering should be clear.

• Paper choice and paper colour

Paper should be thick enough to ensure that there is not a high degree of 'shadowing' from the text over the page. Some material is difficult to read because of this, and letters and words become difficult to distinguish. Obviously thicker paper is more expensive, but it is worth it to get the message across.

Darker colours generally provide more difficult backgrounds for reading. Blue and purple are worse than others.

However, some people with dyslexic tendencies find the contrast of black print on white paper difficult to cope with. They may find text easier to read if it is presented on cream or off-white paper, or even a pale pastel colour. Glossy paper causes glare.

The British Dyslexia Association Style Guide has helpful advice, including recommendations for making on-screen documents and websites easier to read. It can be found here:
http://www.bdadyslexia.org.uk/extra352.html#presentation

2. Readability

To match the text to the abilities of readers, consider these elements.

• Sentence length

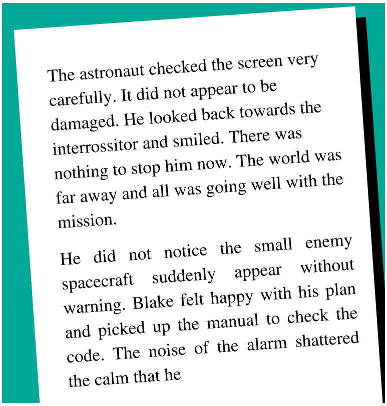
The astronaut checked the screen very carefully. It did not appear to be damaged. He looked back towards the interrossitor and smiled. There was nothing to stop him now. The world was far away and all was going well with the mission.

He did not notice the small enemy spacecraft suddenly appear without warning. Blake felt happy with his plan and picked up the manual to check the code. The noise of the alarm shattered the calm that he

The key to producing clear texts is often the way you write, rather than what you write about. Some subjects involve the use of difficult language and concepts – they can nonetheless be clearly communicated. Sentence length is an extremely important part of this. Sentences (like this one) that run for several lines, with several clauses (and asides including brackets), and that are probably several sentences shoved into one, are not easy for people with reading difficulties. It is far better to write sentences with one or two clauses. It is worth trying to include one main point only in each sentence. Use full stops rather than semi-colons.

Similarly, shorter paragraphs are in general easier to read. Lines and lines of dense print can be difficult for the eye to 'track'. Readers may forget the sense of the beginning of the paragraph by the time they get to the end.

• Choice of words

Some writers have a notion of style that leads them to use a certain kind of jargon. For instance, rather than repeat the word 'said', they will use 'stated', 'revealed', 'declared', 'claimed', etc. In most cases 'said' would do. For readers with

difficulties repetition can be very helpful, particularly with words that may be unfamiliar.

Choosing words that are easier to read is not always an option. All subjects have technical words which need to be read. For example, there is no easy way to write ‘electricity’ – ‘power that comes out of a plug’ is both confusing and inaccurate. A reader with difficulties will need to be taught strategies in order to recognise the word as it appears in a text. Try therefore to use the appropriate word, rather than trying to paraphrase simply.

The passive voice is often more difficult to understand. For example:

‘The screw is placed in the securing hole.’

It may not always be possible to avoid the use of the passive voice, but in general the active voice is easier to understand.

‘Put the screw in the securing hole.’

A noun and adjectives in large clusters are not easy to decode. For example:

‘Middle East hostage release negotiation drama.’

Reading is much easier when the text either matches the ability level of the reader, or is only a little above it. A few simple, quick checks assist in finding out if this is the case.

• Readability tests

You may wish to check the readability of your texts. Readability is an attempt to match the reading level of written material to the 'reading with understanding' level of the reader.

A readability formula carries out calculations on a text, based primarily on sentence and word length, and results in a numerical score. However, other factors affect the understanding of what is being read, which cannot be measured in this way. These include the motivation of the reader, the size and type of print, the layout of written material, previous knowledge of the subject, and the style of the writer.

If you are using a word processor to produce your text there will probably be an option to calculate readability through the spelling and grammar check facility. You will need to tick the box 'show readability statistics' and the readability level will be shown when you have completed your spelling and grammar check.

Different readability formulae are likely to give different scores on the same piece of text, as they give different weightings to various aspects of the text, and may have been developed and tested on different types of text. It's important to use the same formula if you are trying to compare the readability level of a number of different texts.

If you want to check the readability level of a printed text, the simplified version of the SMOG (Simple Measure of Gobbledegook) readability formula shown in the next section is easy to use.[1]

---

1 The original SMOG formula gave a score related to American school grade levels. In the version shown here the constant added at the end has been changed from 3 to 8, to give a readability level.

• SMOG Readability Formula – simplified

SMOG (Simple Measure Of Gobbledegook) is much quicker and easier to work out by hand than other formulae.

1. Select a text
2. Count 10 sentences
3. Count the number of words that have three or more syllables
4. Multiply this by 3
5. Circle the number closest to your answer

1	4	9	16	25	36	49	64	81	100	121	144	169

6. Find the square root of the number you circled

1	4	9	16	25	36	49	64	81	100	121	144	169
1	2	3	4	5	6	7	8	9	10	11	12	13

7. Add 8 → ◯ Readability Level

To obtain the most accurate readability level on longer texts you should carry out this test on three different sections of the document, for example, the beginning, the middle and the end, and take the average of the three scores.

Interpreting the simplified SMOG readability score

You may find it helpful to relate your simplified SMOG result to these average scores for newspaper editorials:

The Sun: under 14

The Daily Express: under 16

The Telegraph and The Guardian: over 17

Alternatively, it may be helpful to relate this readability score to levels in the National Adult Literacy Standards. As a rough indication:

- 9–10 = approx. Entry Level 3;
- 11–12 = approx. Level 1; and
- 14–15 = approx. Level 2.

However, it's far from an exact science and a readability test should only be taken as one indicator among many for the suitability of a text.

Readability tests were developed from normal running text (prose). They are not really suitable for use on text with very different structures such as poetry, labels on bottles or text messages.

First published in 2005. Revised in 2009.

A1880

promoting adult learning
