# Supplementary material for: Web-Based Health Information Following the Renewal of the Cervical Screening Program in Australia: Evaluation of Readability, Understandability, and Credibility
Source: J Med Internet Res. 2020 Jun 26;22(6):e16701. doi: 10.2196/16701 (PMC7381085; doi:10.2196/16701)
Supplement: Multimedia Appendix 3 [file jmir_v22i6e16701_app3.pdf]

Multimedia Appendix: Website URL's and eligibility of all identified websites by search order.

Website URL	Website developer or abbreviation used in manuscript	Included or Excluded	Reason for Exclusion
Google			
http://www.cancerscreening.gov.au/internet/screening/publishing.nsf/Content/cervical-screening-1	*NCSP*	Included	
http://www.cancerscreening.gov.au/internet/screening/publishing.nsf/Content/cervical-screening-1	*NCSP*	Excluded	Duplicate
http://www.health.gov.au/internet/screening/publishing.nsf/Content/cervical-screening-1	*NCSP*	Excluded	Duplicate
https://www.cancer.org.au/about-cancer/early-detection/screening-programs/cervical-cancer-screening.html	*CCA Main Site*	Included	
https://www.cancer.org.au/cervicalscreening/	*CCA Cervical Screening Consumer Site*	Included	
http://www.health.gov.au/internet/screening/publishing.nsf/Content/cervical-screening-1	*NCSP*	Excluded	Duplicate
https://www.healthdirect.gov.au/cervical-screening-test	*Health Direct*	Included	
https://jeanhailes.org.au/health-a-z/health-checks/cervical-screening-test	*Jean Hailes*	Included	
https://www.racgp.org.au/clinical-resources/clinical-guidelines/key-racgp-guidelines/view-all-racgp-guidelines/red-book/early-detection-of-cancers/cervical-cancer	*RACGP*	Included	
https://www.healthywa.wa.gov.au/Articles/A_E/Cervical-Screening-Test	*WA CSP*	Included	
https://www.healthywa.wa.gov.au/Articles/A_E/Cervical-Screening-Test	*WA CSP*	Excluded	Duplicate
https://www.ranzcog.edu.au/RANZCOG_SITE/media/RANZCOG-MEDIA/Women%27s%20Health/Statement%20and%20guidelines/Clinical-Obstetrics/Cervical-cancer-screening-in-Australia-(C-Gyn-19)-Review-July-2017.pdf?ext=.pdf	*RANZCOG*	Excluded	PDF
https://www.cancer.nsw.gov.au/cervical-screening-nsw	*NSW CSP*	Included	
https://www.cancer.nsw.gov.au/cervical-screening-nsw/your-cervical-screening-appointment/what-happens-at-a-cervical-screen	*NSW CSP*	Excluded	Duplicate

https://www.betterhealth.vic.gov.au/health/conditionsandtreatments/cervical-screening-tests	*Victoria CSP*	Included	
https://www.health.qld.gov.au/public-health/cancer-screening/cervical/program	*Queensland CSP (A)*	Included	
Yahoo!			
http://www.cancerscreening.gov.au/internet/screening/publishing.nsf/Content/cervical-screening-1	*NCSP*	Excluded	Duplicate
http://www.cancerscreening.gov.au/internet/screening/publishing.nsf/Content/cervical-screening-1	*NCSP*	Excluded	Duplicate
http://www.health.gov.au/internet/screening/publishing.nsf/Content/cervical-screening-1	*NCSP*	Excluded	Duplicate
https://www.cancer.org.au/about-cancer/early-detection/screening-programs/cervical-cancer-screening.html	*CCA main site*	Excluded	Duplicate
https://www.cancer.org.au/cervicalscreening/	*CCA cervical screening consumer site*	Excluded	Duplicate
https://www.cancer.org.au/about-cancer/early-detection/early-detection-factsheets/understanding-your-pap-smear-results.html	*CCA main site*	Excluded	Duplicate
https://www.healthdirect.gov.au/cervical-screening-test"	*Health Direct*	Excluded	Duplicate
https://www.racgp.org.au/clinical-resources/clinical-guidelines/key-racgp-guidelines/view-all-racgp-guidelines/red-book/early-detection-of-cancers/cervical-cancer	*RACGP*	Excluded	Duplicate
https://jeanhailes.org.au/health-a-z/health-checks/cervical-screening-test	*Jean Hailes*	Excluded	Duplicate
https://www.healthywa.wa.gov.au/Articles/A_E/Cervical-Screening-Test	*WA CSP*	Excluded	Duplicate
https://healthywa.wa.gov.au/Articles/A_E/Cervical-screening	*WA CSP*	Excluded	Duplicate
https://canceraustralia.gov.au/about-us/news/changes-national-cervical-screening-program	*Cancer Australia Cervical Cancer*	Excluded	News release
https://cervical-cancer.canceraustralia.gov.au/screening	*Cancer Australia Cervical Cancer*	Included	
https://www.cancer.nsw.gov.au/cervical-screening-nsw/your-cervical-screening-appointment/what-happens-at-a-cervical-screen	*NSW CSP*	Excluded	Duplicate
https://www.betterhealth.vic.gov.au/health/conditionsandtreatments/cervical-screening-tests	*Victoria CSP*	Excluded	Duplicate
Bing			
http://cancerscreening.gov.au/internet/screening/publishing.nsf/Content/cervical-screening-1	*Cancer Screening*	Excluded	Duplicate

https://www.cancer.org.au/cervicalscreening/	*CCA cervical screening consumer site*	Excluded	Duplicate
http://www.cancerscreening.gov.au/internet/screening/publishing.nsf/Content/healthcare-providers	*NCSP*	Excluded	Duplicate
https://www.healthdirect.gov.au/cervical-screening-test	*Health Direct*	Excluded	Duplicate
https://www.betterhealth.vic.gov.au/health/conditionsandtreatments/cervical-screening-tests	*Victoria CSP*	Excluded	Duplicate
https://www.health.qld.gov.au/healthsupport/businesses/pathology-queensland/healthcare/testing/cervical-screening	*Queensland CSP (B)*	Included	
https://www.cancer.org.au/about-cancer/early-detection/screening-programs/cervical-cancer-screening.html	*CCA main site*	Excluded	Duplicate
https://learn.nps.org.au/mod/page/view.php?id=7804	*NPS MedicineWise*	Included	
https://www.cancer.nsw.gov.au/cervical-screening-nsw	*NSW CSP*	Excluded	Duplicate
https://en.wikipedia.org/wiki/Cervical_screening	*Wikipedia*	Excluded	Wikipedia
https://wiki.cancer.org.au/australia/Guidelines:Cervical_cancer/Screening	*NCSP clinical guidelines*	Included	
https://www.health.qld.gov.au/public-health/cancer-screening/cervical/program	*Queensland CSP (A)*	Excluded	Duplicate
https://learn.nps.org.au/mod/page/view.php?id=7804	*NPS MedicineWise*	Excluded	Duplicate
https://www.ncsr.gov.au/	*NCSR*	Included	
http://www.cervicalscreen.health.gov.au/	*NCSP*	Excluded	Duplicate
https://www.cancer.nsw.gov.au/cervical-screening-nsw/about-cervical-screening/how-has-cervical-screening-changed	*NSW CSP*	Excluded	Duplicate
http://www.health.gov.au/internet/screening/publishing.nsf/Content/frequently-asked-questions-3	*NCSP*	Excluded	Duplicate
http://www.cervicalscreen.health.gov.au/	*NCSP*	Excluded	Duplicate

NCSP: National Cervical Screening Program, CCA: Cancer Council Australia, RACGP: Royal Australian College of General Practice, WA: Western Australia, CSP: Cervical Screening Program, RANZCOG: Royal Australian and New Zealand College of Obstetricians and Gynaecologists NSW: New South Wales, NPS: National Prescribing Service, NCSR: National Cancer Screening Register.

This is a Multimedia Appendix to a full manuscript published in the J Med Internet Res. For full copyright and citation information see http://dx.doi.org/10.2196/jmir.16701
