# Supplementary material for: Web-Based Health Information Following the Renewal of the Cervical Screening Program in Australia: Evaluation of Readability, Understandability, and Credibility
Source: J Med Internet Res. 2020 Jun 26;22(6):e16701. doi: 10.2196/16701 (PMC7381085; doi:10.2196/16701)
Supplement: Multimedia Appendix 4 [file jmir_v22i6e16701_app4.pdf]

Multimedia Appendix: Website abbreviation, organisation and target audience of included websites.

Website abbreviation	Organisation	Target Audience
NCSP	*Australian Government Department of Health*	*Consumers and Healthcare providers*
CCA Cervical Screening Consumer Site	*Cancer Council Australia*	*Consumers*
Health Direct	*Health Direct*	*Consumers*
Jean Hailes	*Jean Hailes*	*Consumers*
RACGP	*RACGP*	*Healthcare providers*
WA CSP	*Government of Western Australia*	*Consumers*
NSW CSP	*Cancer Institute NSW Government of New South Wales*	*Consumers and Healthcare providers*
Victoria CSP	*Government of Victoria*	*Consumers*
CCA[b] Main Site	*Cancer Council Australia*	*Consumers*
Queensland CSP (A)	*Government of Queensland*	*Consumers*
Cancer Australia Cervical Cancer	*Australian Government Cancer Australia*	*Consumers*
Queensland CSP (B)	*Government of Queensland*	*Healthcare providers*
NPS MedicineWise	*NPS MedicineWise*	*Healthcare providers*
NCSP clinical guidelines	*Cancer Council Australia*	*Healthcare providers*
NCSR	*Australian Government Department of Health*	*Healthcare providers*

NCSP: National Cervical Screening Program, CCA: Cancer Council Australia Royal Australian College of General Practitioners, CSP: Cervical Screening Program, NPS: National Prescribing Service, NCSR: National Cancer Screening Register.

This is a Multimedia Appendix to a full manuscript published in the J Med Internet Res. For full copyright and citation information see http://dx.doi.org/10.2196/jmir.16701
